# Facile Graphene Oxide Modification Method via Hydroxyl-yne Click Reaction for Ultrasensitive and Ultrawide Monitoring Pressure Sensors

**DOI:** 10.1021/acsami.3c17172

**Published:** 2024-01-26

**Authors:** Zhipeng Hu, Wanlong Lu, Youbin Zheng, Jiamei Liu, Hossam Haick, Laju Bu

**Affiliations:** † School of Chemistry, Engineering Research Center of Energy Storage Materials and Devices, Ministry of Education, Xi’an Key Laboratory of Sustainable Energy Material Chemistry, Xi’an Jiaotong University, Xi’an, Shaanxi 710049, P. R. China; ‡ Department of Chemical Engineering and Russell Berrie Nanotechnology Institute, Technion-Israel Institute of Technology, Haifa 3200003, Israel; § Department of Electrical Engineering and Electronics, University of Liverpool, Liverpool L69 3GJ, U.K.; ∥ Instrumental Analysis Center, Xi’an Jiaotong University, Xi’an, Shaanxi 710049, P. R. China

**Keywords:** GO modification, hydroxyl-yne click reaction, GO-PDMS sponge, pressure sensors, wearable electronics

## Abstract

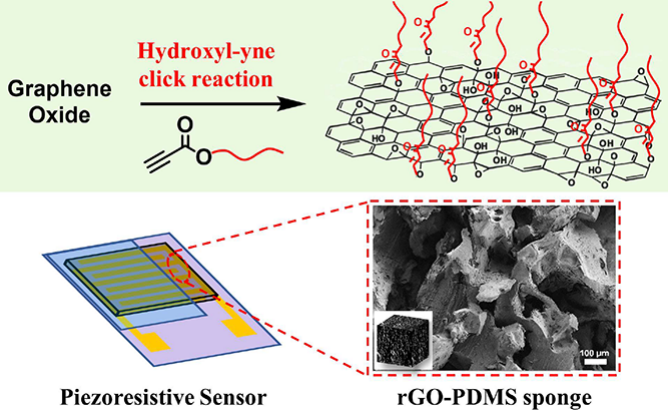

Enhancing the durability and functionality of existing materials through sustainable pathways and appropriate structural design represents a time- and cost-effective strategy for the development of advanced wearable devices. Herein, a facile graphene oxide (GO) modification method via the hydroxyl-yne click reaction is present for the first time. By the click coupling between propiolate esters and hydroxyl groups on GO under mild conditions, various functional molecules are successfully grafted onto the GO. The modified GO is characterized by FTIR, XRD, TGA, XPS, and contact angle, proving significantly improved dispersibility in various solvents. Besides the high efficiency, high selectivity, and mild reaction conditions, this method is highly practical and accessible, avoiding the need for prefunctionalizations, metals, or toxic reagents. Subsequently, a rGO-PDMS sponge-based piezoresistive sensor developed by modified GO-P2 as the sensitive material exhibits impressive performance: high sensitivity (335 kPa^–1^, 0.8–150 kPa), wide linear range (>500 kPa), low detection limit (0.8 kPa), and long-lasting durability (>5000 cycles). Various practical applications have been demonstrated, including body joint movement recognition and real-time monitoring of subtle movements. These results prove the practicality of the methodology and make the rGO-PDMS sponge-based pressure sensor a real candidate for a wide array of wearable applications.

## 1 Introduction

In the pursuit of carbon neutrality and a sustainable future, it is imperative to investigate advanced materials that produce minimal or even zero carbon emissions while maintaining high performance.^1−5^ While there is no doubt about the importance of researching and developing new ecofriendly materials, it is essential to acknowledge the time-consuming and costly nature of this process. As an alternative to developing novel materials, enhancing the robustness, durability, and functionality of existing materials offers a feasible strategy for achieving our sustainability objectives. On the other hand, significant attention has been dedicated to wearable electronics,^6−11^ which play a pivotal role in advancing motion recognition and detection,^12,13^ human–machine interaction,^14,15^ and intelligent robotics.^16^ Exploring innovative sensitive materials and structural designs represents a time and cost-efficient approach to developing advanced wearable devices with properties of high sensitivity, wide detection range, and enhanced stability and durability.^6,17−19^ Various sensitive materials have been documented, including zero-dimensional metal nanoparticles,^20^ one-dimensional conductive polymer fibers and metal nanowires,^21,22^ as well as two-dimensional graphene and graphene-like materials.^23,24^

Graphene materials are widely regarded as among the most promising options for high-performance devices due to their exceptional electrical conductivity, thermal conductivity, and mechanical stability.^25−31^ Various GO-based composite films,^25^ papers,^27^ foams,^32^ and sponges^33^ have demonstrated excellent performance in the context of wearable pressure sensors. However, limited by GO’s inherent poor dispersion, poor processability, and monofunctionality, endowing GO-based pressure sensors with high sensitivity to small and large deformations to meet the growing demand for high sensitivity versatility and wide range testing requirements remains a great challenge.^34−36^ Consequently, the chemical modification of graphene to enhance its dispersion, processability, and multifunctionality has emerged as an appealing approach to broaden its practical applicability.

**Figure 1:**
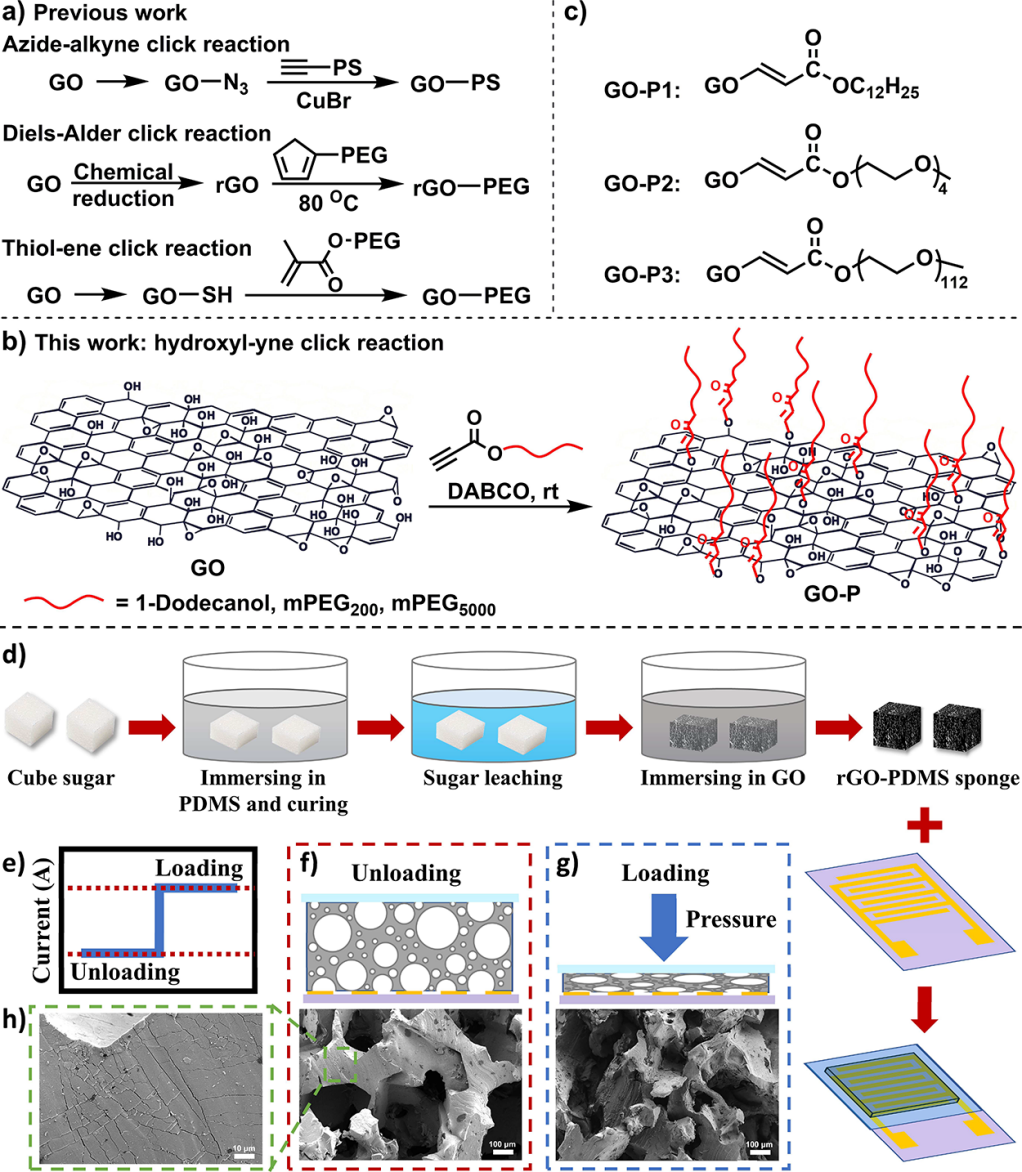
Overview of GO modification methods via click reactions: (a) previous works about GO modification, (b) GO modification via hydroxyl-yne click reaction, and (c) corresponding GO-P products in this work. (d) Schematic diagram of the fabrication process of the rGO-PDMS sponge-based pressure sensor. Schematic illustration of the sensing mechanism: (e) current response, the SEM images of the sensing layer (f) in the initial state and (g) under pressure, and (h) wrinkle structure on the surface of GO.

One of the most effective strategies for functionalizing GO is the grafting of chemical groups using click chemistry, attributed to its significant advantages such as high efficiency, selectivity, robustness, and functional group tolerance.^37−39^ Substantial endeavors have been devoted to implement a series of click reactions (e.g., Cu(I)-catalyzed azide–alkyne click reaction,^40,41^ Diels–Alder click reaction,^42^ and thiol–ene click reaction^43^) by anchoring reactive sites such as aryl rings, double bonds, and hydroxyl groups on GO, and a variety of GO modification methods have been developed (Figure 1a).^34,36,44−47^ These strategies successfully modified functional groups on GO, such as polystyrene, polyethylene glycol, functionalized aryl, polyethylene brushes, etc., which significantly improved the dispersion of GO in common solvents and enhanced its functionality. Nonetheless, it comes with several drawbacks, including high reaction temperature, catalyst residues and postprocessing difficulties, the use of metals and toxic reagents, the need for premodification, etc., which are contrary to the principles of green chemistry and have greatly limited their applications. In particular, addressing the intrinsic challenges of poor dispersibility and complex postprocessing of GO, there is a pressing need for the development of a more sustainable, facile, and accessible strategy for GO modification.

In 2014, Fernando reported an impressive click reaction targeting hydroxyl groups, known as the Lewis base-catalyzed hydroxyl-yne click reaction,^48^ which exhibited great advantages of high efficiency, mild reaction conditions, simple postprocessing, and improved sustainability. Subsequently, the hydroxyl-yne click reaction was developed into the field of polymers by Tang and co-workers.^49^ The efficient polymerization of aromatic and aliphatic diols with diynes at room temperature^50,51^ and the etherification of cellulosic materials^52,53^ under similar conditions have been sequentially documented, affirming the suitability of hydroxyl groups in polymers for the hydroxyl-yne click reaction. Given the abundance of multiple hydroxyl groups on GO,^35^ it holds significant potential for modifications through the hydroxyl-yne click reaction. However, to the best of our knowledge, no relevant studies have been reported on this subject thus far.

In this work, a facile GO modification strategy was developed via the hydroxyl-yne click reaction between the propiolates and hydroxyl groups on GO, enabling successful grafting of various functional molecules onto GO. Besides the advantages of high efficiency, exceptional selectivity, and mild reaction conditions, this method was highly practical and accessible. The direct utilization of commercial GO without prior functionalization and the use of organic small-molecule catalysts instead of metal or toxic reagents ensured simplicity and operable postprocessing manipulation, which were crucial for practical applications. The success of the modification was confirmed through FTIR, XRD, TGA, XPS, etc. Furthermore, the efficacy of this strategy was further underscored by the substantial enhancement of the dispersion and stability of the modified GO in common solvents. Three-dimensional and porous conducting sponges have emerged as promising candidates for flexible pressure sensors, demonstrating excellent superiority.^29,30,54^ Subsequently, the rGO-PDMS sponge-based piezoresistive sensor was developed, utilizing the modified GO-P2 as the sensitive material, which exhibited exceptional detection range and sensitivity for pressure and deformation stimuli, as well as maintaining excellent stability after >5000 pressure loading tests. A range of practical wearable applications were then explored, including body joint motion recognition (finger joints, wrist joints, articulated elbows, neck movements), real-time monitoring of subtle movements (muscle movements, swallowing, respiration, and carotid artery pulse), and circuit switching control via voltage modulation. This innovative strategy provides a robust and efficient alternative for developing new advanced functional materials, and the rGO-PDMS sponge-based pressure sensor turns out to be a promising candidate for a diverse range of wearable technology applications.

## 2 Experimental Section

### 2.1 Preparation of Propiolate Esters

In a round-bottom flask, propargyl acid **1** (10 mmol), hydroxyl substrate **2** (11 mmol), solvent toluene (40 mL), and catalyst *p*-toluenesulfonic acid (1 mmol) were added sequentially. The reaction was then refluxed for 8 h. After completion of the reaction, the target product propiolate esters **3** was purified by column chromatography or precipitation.

### 2.2 Hydroxyl-yne Click Reaction

First, GO was dispersed in DMF (5 mg/mL) with the assistance of sonication. Then, the catalyst DABCO (20%) was added, followed by the dropwise addition of propiolate esters in batches. The reaction mixture was then stirred for 3 h at room temperature. The mixture was dialyzed with water or centrifuged to yield the modified GO-P.

### 2.3 Preparation of a PDMS Sponge

First, the PDMS main agent and curing agent were mixed at a mass ratio of 10:1, then the template cube sugar was immersed and vacuum pumped for 1 h to remove air bubbles, and the samples were cured at 80 °C for 3 h. The cube sugar was subsequently removed using hot water at 100 °C, and the resulting material was dried in a vacuum oven at 60 °C for 3 h to obtain a PDMS sponge.

### 2.4 Preparation of a rGO-PDMS Sponge

The prepared PDMS sponges were treated with plasma (oxygen, 10 mL of SCCM, 1 min). Then they were immersed into an ethanol solution of APTES (5 mmol/mL) for 2 h at room temperature. The modified sponges were washed with DDW and dried in a vacuum oven at 60 °C for 3 h. The modified PDMS sponge was immersed in a GO-P2/DDW dispersion (2 mg/mL) for 2 h. After the dip-coating process, the sponge was dried at 60 °C for 5 h to produce the GO-PDMS sponge. Subsequently, it underwent reduction using hydrazine hydrate vapor at 70 °C for 2 h, followed by washing with DDW and drying to yield the rGO-PDMS sponge.

### 2.5 Preparation of a rGO-PDMS Sponge-Based Piezoresistive Sensor

Gold interdigital electrodes were prepared on polyimide films by shadow-mask vaporization. The rGO-PDMS sponge sensing layer was placed on the electrode, and two copper wires were connected by conductive silver paste. Finally, after packaging with the PE film, the rGO-PDMS sponge-based piezoresistive sensor was successfully prepared. The dimensions of the rGO-PDMS sponge are 10 mm × 10 mm × 2.5 mm (length × width × thickness). The specific parameters of the interdigital electrode are illustrated in Figure S6.

### 2.6 Characterizations and Measurements

The morphology and composition of the samples were characterized using scanning electron microscopy (SEM, Gemini SEM 500), X-ray diffraction (XRD, Bruker D8 ADVANCE), X-ray photoelectron spectroscopy (XPS, Thermo Fisher ESCALAB Xi+), a Fourier-transform infrared spectrometer (FTIR, Nicolet iS10), an optical contact angle measurement instrument (KRUSS DSA100S), and a simultaneous thermal analyzer (TGA, METTLER TOLEDO TGA/DSC3+). Pressure and bending were loaded by using a homemade automation module, and the current or voltage signals from the sensors were recorded by a digital electrometer (Agilent B2900A).

## 3 Results and Discussion

### 3.1 GO Modification via Hydroxyl-yne Click Reaction and Characterizations

As shown in Scheme S1, first a series of propiolate esters were prepared via the well-standardized procedures. Subsequently, commercial GO and prepared propiolate esters were subjected to a hydroxyl-yne click reaction catalyzed by DABCO at room temperature. After 5 h of reaction, a series of modified GO materials were successfully prepared, referred to as GO-P. GO-P1, GO-P2, and GO-P3 were prepared from dodecyl propiolate, mPEG_200_ propiolate, and mPEG_5000_ propiolate, respectively. According to reports, the reaction mechanism of this chemical process is illustrated in Scheme S2.^48,53^ This process may generate byproduct related to propiolates, but it can be effectively suppressed through appropriate operational procedures. Additionally, residual impurities (such as propiolate, catalyst, and byproducts, etc.) can be easily removed through centrifugation or dialysis. The success of the click coupling was confirmed through various tests, including FTIR, XRD, TGA, contact angle measurements, and assessment of the dispersion of the product in common solvents.

**Figure 2:**
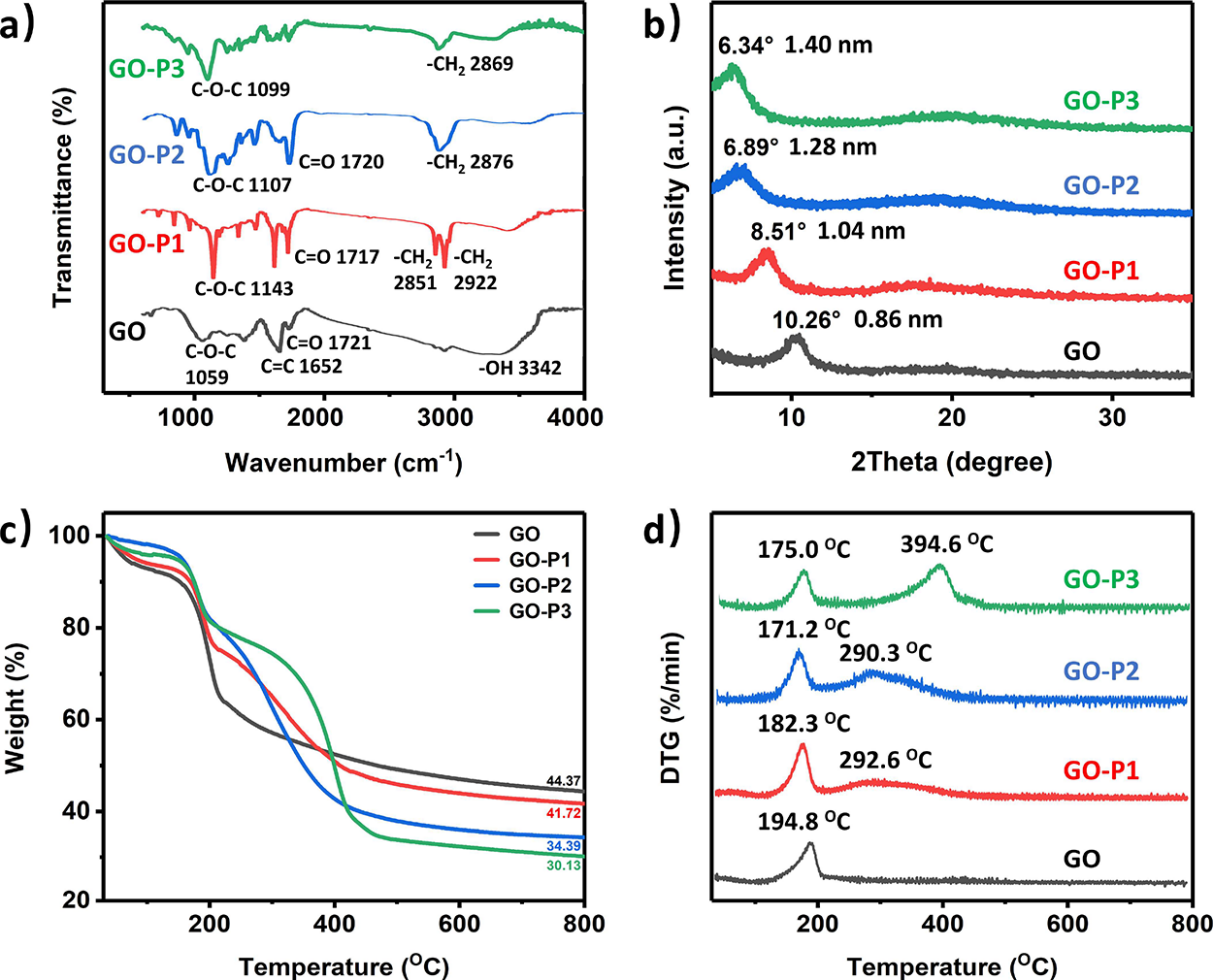
(a) FTIR spectra, (b) XRD patterns, and (c) TGA and (d) DTG curves of GO, GO-P1, GO-P2, and GO-P3.

The FTIR spectra depicted in Figure 2a were utilized to qualitatively verify the modification of GO. In the spectrum of pristine GO, the significant band at 3342 cm^–1^ corresponded to the −OH of GO. Additionally, bands at 1721, 1652, and 1059 cm^–1^ were attributed to the stretching vibrations of C=O, C=C, and C–O–C on GO, respectively. Notably, the peaks in the spectra of the modified GO-P materials exhibited significant changes in comparison to those of pristine GO. The absorption intensity at 3342 cm^–1^ was notably reduced, indicating substantial consumption of hydroxyl groups during the click reaction. Furthermore, several new characteristic bands emerged, obviously derived from the grafted propiolates. For instance, in the IR spectra of GO-P1, two distinct peaks appeared at 2922 and 1143 cm^–1^, corresponding to the C–H and C–O–C stretching vibrations of the grafted alkyl chain and ester group, respectively. Similarly, in the IR spectra of GO-P2, two prominent absorption peaks were observed at 2876 and 1107 cm^–1^, attributed to the C–H and C–O–C stretching vibrations of the grafted mPEG chains. In addition, an enhanced absorption associated with C=O of the ester group was observed at 1720 cm^–1^. GO-P3 exhibited a similar trend to that of GO-P2.

The XRD plot presented in Figure 2b revealed alterations in the interlayer spacing, clearly indicating the preservation of an organized layer structure in the modified GO material. The X-ray peak of pristine GO was observed at 2θ = 10.26°, corresponding to an interlayer distance of 0.86 nm. After graft modification, the peak of GO-P1 shifted to 8.51°, signifying an increased layer thickness of 1.04 nm. Likewise, the peak for GO-P2 shifted to 6.89°, reflecting a layer thickness of 1.28 nm, and the peak for GO-P3 shifted to 6.34°, indicating a layer thickness of 1.40 nm. In contrast to the precursor GO, the modified GO-P materials exhibited a substantial increase in the layer spacing. This change could be attributed to the grafting of molecular chains onto the surface of GO, resulting in a reduction in the stacking compactness of the GO sheets, thereby further increasing the interlayer spacing between the sheets.

Thermogravimetric analysis (TGA) provided valuable insights into the composition and thermal stability of the samples (Figures 2c,d and S1). In all cases, a weight loss was observed at around 100 °C, attributed to the loss of interstitial water. The pristine GO showed one significant weight loss peak at 194.8 °C, primarily associated with the pyrolysis of oxygen-containing groups, and it underwent complete decomposition at around 750 °C, leaving a residual carbon of 44.37%. In contrast, the thermal analysis patterns of the modified GO materials markedly differed from those of pristine GO. GO-P1, GO-P2, and GO-P3 exhibited two distinct weight losses: the first peak occurs, respectively, at 182.3, 171.2, and 175.0 °C, close to the original GO data; and the second peak occurs, respectively, at 292.6, 290.3, and 394.6 °C, attributed to the pyrolysis of the grafted molecular chains and also completely decomposed at around 750 °C (residual carbon ratios of 41.72, 34.39, and 30.13%, respectively). The calculated grafting ratios for GO-P1, GO-P2, and GO-P3 were 2.65, 9.98, and 15.21%, respectively, and the actual values should be larger considering the coking residues of the polymers. Additionally, the grafting ratio could be adjusted by regulating the proportion of propiolate ester added (Figure S2).

**Figure 3:**
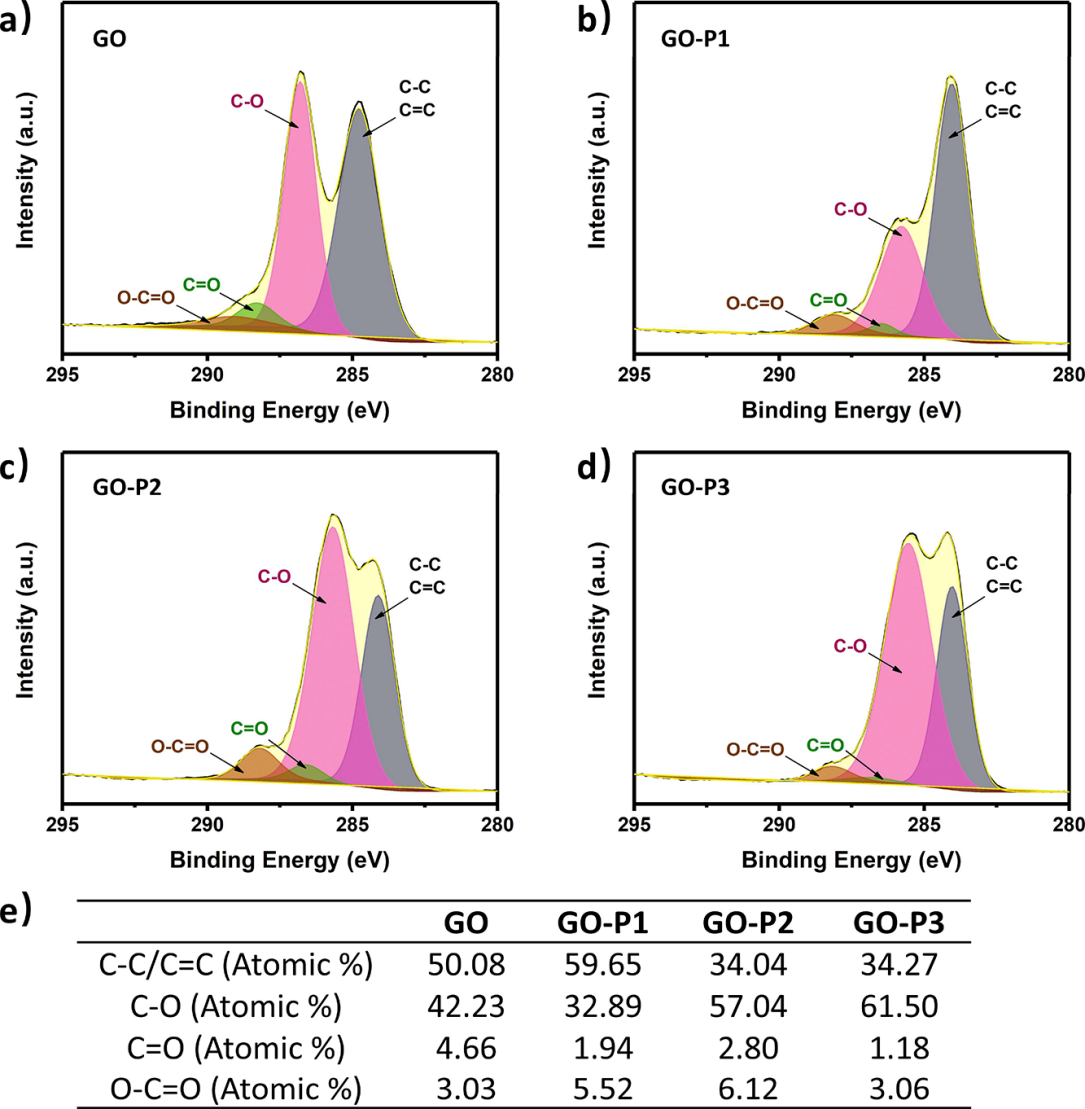
High-resolution XPS spectra of (a) GO, (b) GO-P1, (c) GO-P2, and (d) GO-P3 and (e) corresponding component analysis.

To further demonstrate this hydroxyl-yne click modification strategy, XPS was employed for samples GO, GO-P1, GO-P2, and GO-P3 (Figures 3 and S3). Consistent with prior reports,^42^ the C 1s XPS spectra of GO exhibited four distinctive peaks, each corresponding to different carbon components within various functional groups: C–C/C=C (284.2 eV), C–O (285.7 eV), C=O (286.6 eV), and C=O (288.2 eV). Comparing with GO, the other three samples maintained the same composition of these four characteristic peaks but with significantly different intensities. For example, for GO-P1 modified with dodecyl propiolate, the intensity of the C–C/C=C peak was significantly enhanced, indicating the presence of dodecyl alcohol introduced through grafting. Similarly, for GO-P2 and GO-P3, modified with mPEG ester, there was a notable enhancement in the C–O intensity, reflective of the mPEG moiety.

**Figure 4:**
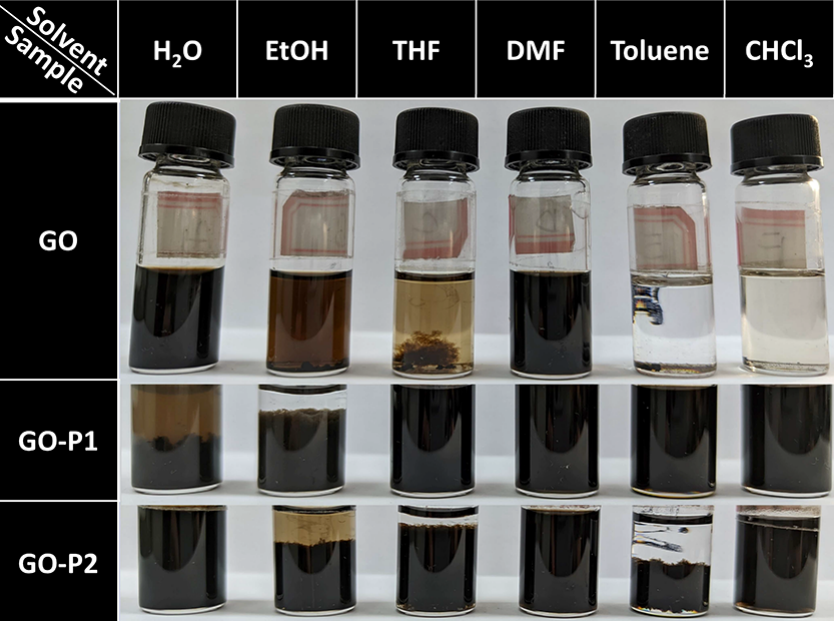
Dispersion of **GO**, **GO-P1**, and **GO-P2** in various solvents (images were taken after one month of sample preparation at a concentration of 1 mg/mL).

Subsequently, an evaluation of the dispersion properties of GO, GO-P1, and GO-P2 was conducted in various common solvents (Figures S4 and 4). Sample photographs are shown in Figure 4, one month after subjecting the materials to sonication dispersion at a concentration of 1 mg/mL. GO exhibited excellent dispersion stability in water and DMF, maintaining a homogeneous dispersion after one month. In contrast, hydrophobically modified GO-P1 exhibited notably reduced dispersibility in water but excellent dispersibility in THF, DMF, toluene, and CHCl_3_. For hydrophilically modified GO-P2, it exhibited excellent dispersibility not only in water and DMF but also in CHCl_3_ when compared with GO. Additionally, significantly improved dispersibility was observed in ethanol, THF, and toluene. It was further confirmed by the water contact angle test (Figure S5). The significant improvement or reversal of the dispersibility was attributed to the effect of covalently grafted molecules on the GO. On one hand, it was influenced by the inherent solubility of the grafted molecules in different solvents; on the other hand, the introduction of grafted molecules on the GO surface weakened the π–π stacking and van der Waals interactions and thus enhanced the dispersion properties.

The significant strengths of this strategy robustly support the method’s potential for widespread commercial adoption in real-world applications, aligning with a key objective in materials science research. Nevertheless, the journey of transitioning a novel methodology from the laboratory to industrial-scale production and eventual market integration is complex and demanding. Particularly, given the inherent sensitivity and processing challenges associated with graphene materials, maintaining control over reaction conditions and ensuring product uniformity in large-scale manufacturing poses a challenge that warrants careful consideration. This emphasizes the need for us to intensify our efforts to adeptly tackle these challenges.

### 3.2 Applications in the rGO-PDMS Sponge-Based Pressure Sensor

Then, a highly sensitive and wide-detection-range rGO-PDMS sponge-based piezoresistive sensor was prepared, with modified GO-P2 as the sensing material. As shown in Figure 1d, the PDMS sponge was initially prepared using sugar cubes as sacrificial templates. Subsequently, rGO-PDMS sponge was prepared through sequential processes involving plasma and APTES surface treatment, dip-coating, and reduction. The rGO-PDMS sponge was then assembled onto a PI substrate with interdigital electrodes, and wires were connected to create piezoresistive sensors. This sensor exhibited outstanding performance, including high sensitivity (335 kPa^–1^, 0.8–150 kPa), wide linear range (>500 kPa), low detection limit (0.8 kPa), and remarkable long-lasting durability (>5000 cycles).

The sensing mechanism of the rGO-PDMS sponge-based piezoresistive sensor is shown in Figure 1e: When pressure loading was applied, the sponge-based sensing layer underwent compressive deformation, creating more conductive pathways, which electrically manifested as a decrease in the device’s resistance, effectively translating mechanical stimuli into electrical signals (resistance, current, and voltage).^25,54^ Specifically, the sensing process included four aspects: (1) the contact points between the sensing layer and the electrodes, which increased when pressure was applied due to the uneven surface of the sensing layer (Figures 1 and S7).^55^ (2) Surface wrinkle structures of rGO as shown in Figure 1h. Constructing wrinkle structures on the GO surface of the sensing layer could significantly improve sensitivity, detection range, and stability of the sensors.^56^ (3) Hollow holes and microbumps inside the sponge (Figure 1e). While loading pressure to the device, the internal holes of the sensing layer were compressed, creating more conductive pathways inside. (4) Interdigital electrodes provided more contact points between the sensing layer and the electrodes compared to two electrodes. These features ensured high sensitivity, wide detection range, low detection limit, and high stability of the piezoresistive sensor.

### 3.3 Performance of the Sensor

**Figure 5:**
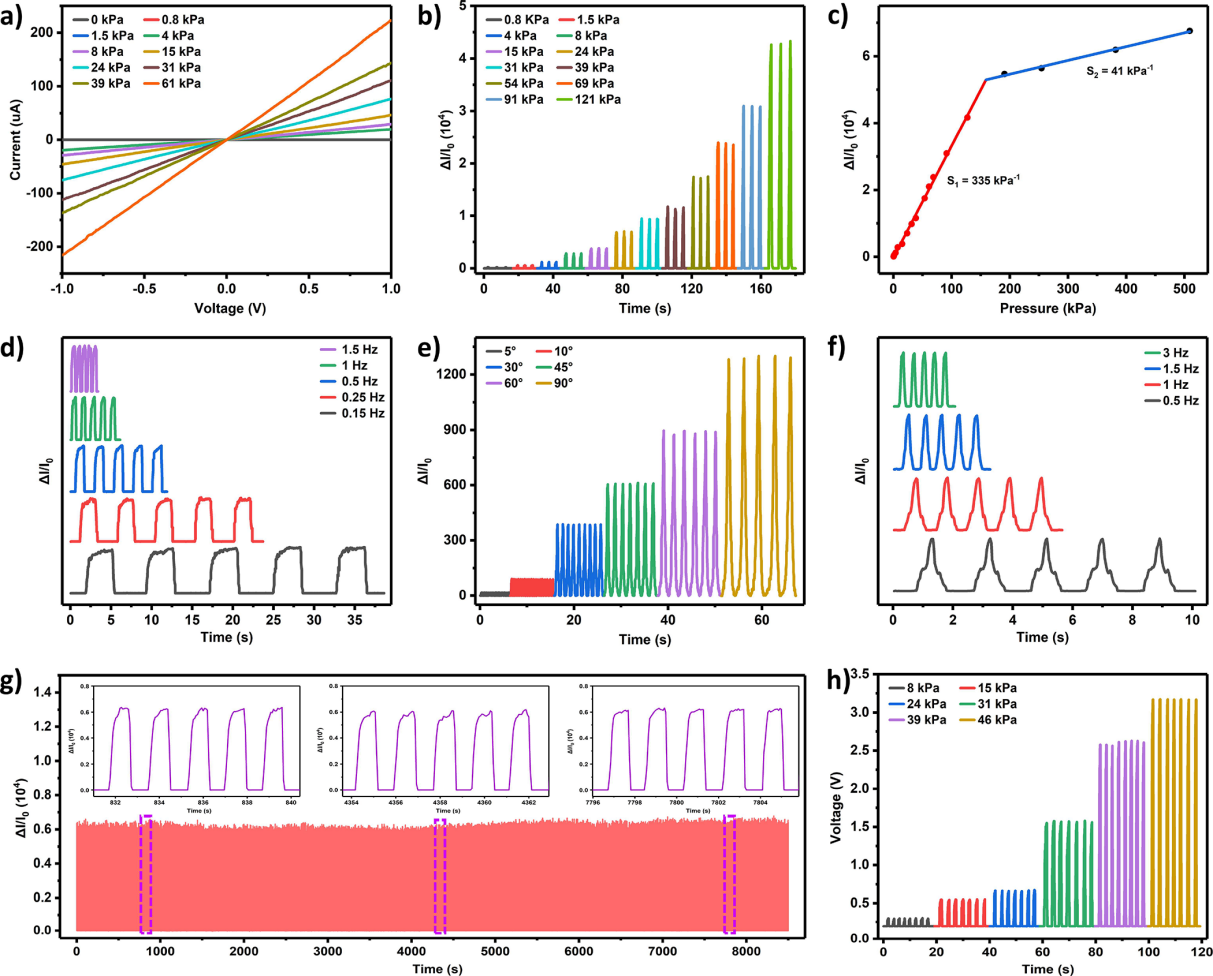
(a) *I*–*V* and (b) *I*–*T* curves of the sensor under different pressures. (c) Sensitivity of the sensor. (d) *I*–*T* curves of the sensor under 15 kPa pressure with different frequencies. The *I*–*T* curves of the sensor under (e) different bending angles and (f) 30° bend with different driving frequencies. (g) Durability performance of the sensor under ∼20 kPa for over 5000 cycles. (h) Voltage modulation under different loading pressures.

The fundamental piezoresistive properties of the sensor were systematically investigated. The linear *I*–*V* curves of the sensor under different pressures shown in Figure 5a indicated good ohmic contact between the sensing layer and electrodes with no impediments affecting charge transfer. The slope of the *I*–*V* curve increased with rising pressure, signifying the formation of more rGO conductive pathways and a higher signal strength in the *I*–*T* curve (Figure 5b). Figure 5c displays the relative change in current (Δ*I*/*I*_0_) with varying pressure loading. These values determine the sensitivity (*S*) of the sensor:
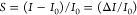
where *I* and *I*_0_ are the instantaneous and unloading current values, respectively. For pressure unloading–loading testing of the sensor, the typical actual unloading current is around 10^–10^. However, when calculating sensitivity, we use a value of 1 × 10^–9^ as the unloading current to eliminate the influence of noise and ensure more reliable results. Naturally, this approach results in a calculated sensitivity that is lower than the actual sensitivity. The sensitivity S_1_ of the sensor was 335 kPa^–1^ (<150 kPa). As the loading pressure increased, the sensitivity of S_2_ decreased to 41 kPa^–1^, but the detection range reached 500 kPa. The output performance of the sensor showed great stability at different loading frequencies (Figures 5d and S8). Furthermore, the sensor’s response to bending deformation was examined. As the bending angle increased, the device’s resistance decreased, leading to a higher signal strength in the *I*–*T* curve (Figure 5e). The current signal at 30° bending under different driving frequencies in Figure 5f also indicated good stability. Additionally, the durability of the rGO-PDMS sponge-based pressure sensor was tested after >5000 pressure loading–unloading cycles at 20 kPa, showing no significant fluctuations in its response and demonstrating the sensor’s excellent stability (Figure 5g). The intrinsic response of the rGO-PDMS sponge-based pressure sensor to pressure and deformation was the change in resistance. In addition to output of the current signal, it could also generate a voltage signal by a signal conversion module. The output voltage signal increased as the loading pressure increased (Figure 5h). This property could find applications in voltage modulation or switching. Based on the presented data, the developed rGO-PDMS sponge-based pressure sensor demonstrated excellent performance, comparable to that of other high-performance sensors reported in the literature (Table S1).

### 3.4 Practical Applications

**Figure 6:**
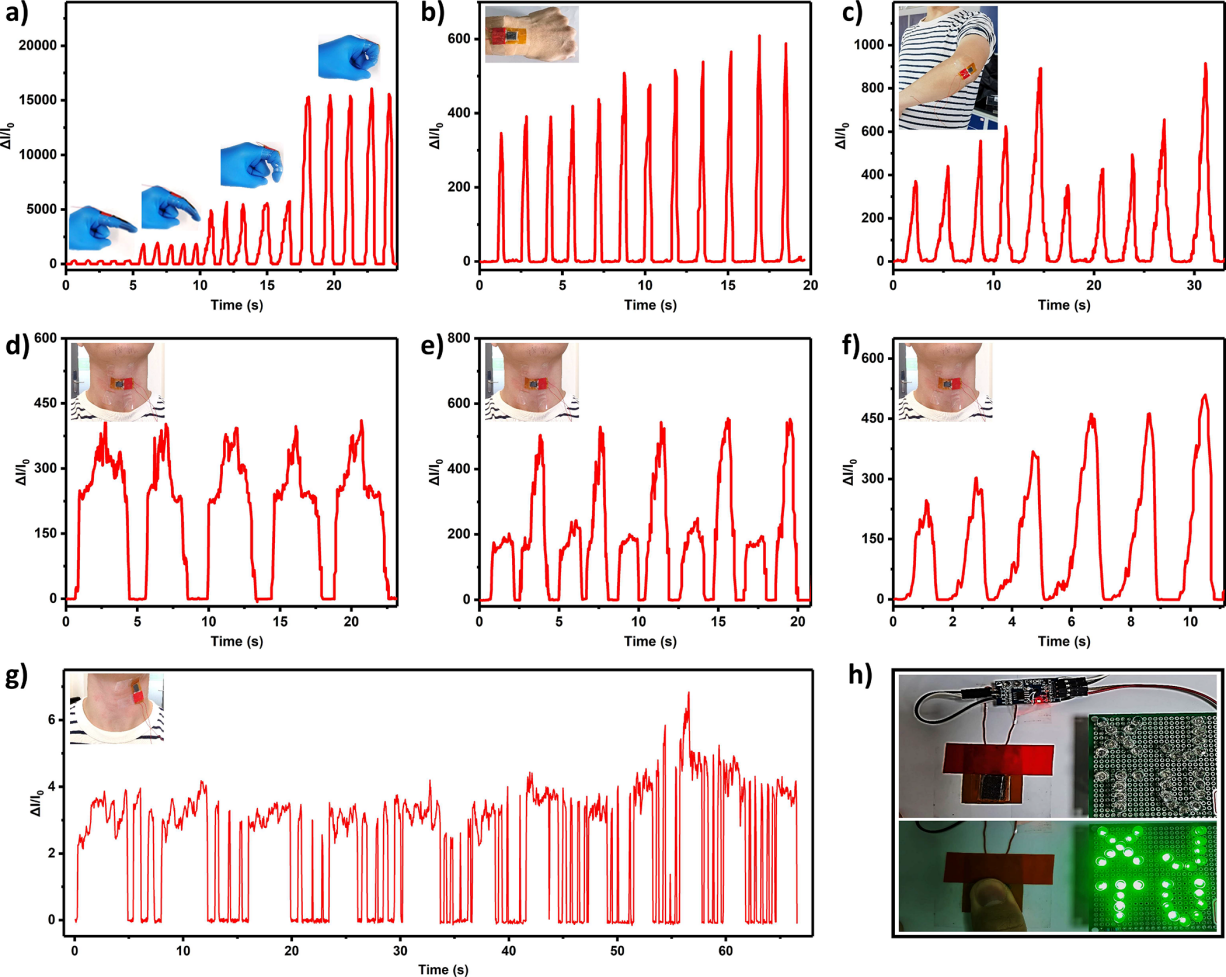
Practical applications of the rGO-PDMS sponge-based piezoresistive sensor. Monitoring of human (a) phalangeal, (b) wrist, and (c) elbow joint bending; (d) hyperextension and (e) flexion of the head motion; (f) throat swallowing motion; (g) real-time monitoring of carotid artery and respiration. (h) Circuit photo of LEDs controlled by a pressure switch.

Practical applications of the rGO-PDMS sponge-based pressure sensor were investigated, including human movement recognition, real-time monitoring, and voltage regulation switching. First, the practicality of the sensor was examined as a flexible device for monitoring the flexion and extension angles of various joints (finger, wrist, and elbow joints, Figure 6a–c). The signal intensity increased with the increasing flexion angles of the joints, enabling the monitoring of joint movements by comparing the signal intensities to ensure that the joints moved at the correct angles. As one of the most vital part of the human body, information about the movements of the neck was crucial, involving breathing, carotid artery pulsation, throat, and head motions. When the sensor was positioned on the throat, it accurately detected and recorded hyperextension (Figure 6d), head flexion (Figure 6e), and swallowing movements (Figure 6f). Furthermore, the versatility of the rGO-PDMS sponge-based sensor allowed real-time monitoring of subtle movements with the high sensitivity of the sensor (335 kPa^–1^, <150 kPa). When the sensor was placed near the carotid artery on the neck, the carotid artery and respiratory signals could be monitored in real time from a person’s resting state to strenuous physical activity (Figure 6g). At rest, the heart rate was 65 beats per minute, and respiration was at 8 breaths per minute (Figure 6g, 0–30 s). As exercise progressed, the heart rate and respiration rates increased to 120 beats and 11 breaths per minute (Figure 6g, 30–65 s), respectively. Furthermore, muscle movement is another type of subtle yet widely prevalent human body movement. Figure S10 demonstrates that the sensor accurately monitored subtle muscle movements, including those of the masticatory muscle, triceps brachii muscle, forearm extensor, and tibialis anterior muscle. These results, in alignment with the device’s design strategy, demonstrated consistency with actual physiological signals and movements. Additionally, the rGO-PDMS sponge-based piezoresistive device could serve as a voltage regulator and act as a circuit switch in response to pressure changes, providing an intuitive means of monitoring pressure levels (Figure 6h).

## 4 Conclusions

In summary, we have successfully developed a facile GO modification method using the hydroxyl-yne click reaction for the first time. Various propiolate esters were click-coupled with the hydroxyl groups on GO, successfully grafting functional molecules onto GO. The modification was validated through various characterization tests, including FTIR, XRD, TGA, XPS, and contact angle measurements. The dispersibility of the modified GO-P materials in common solvents was significantly improved. Compared to previous reports, this approach not only offered advantages such as high efficiency, high selectivity, and robust and mild reaction conditions but also demonstrated high processability. It utilized commercial GO directly without prior functionalization and employed an organic catalyst instead of metals or toxic reagents, ensuring simplicity and operable postprocessing manipulation. Subsequently, an rGO-PDMS sponge-based piezoresistive sensor with ultrahigh sensitivity and ultrawide detection range was developed using the modified GO-P2 as the sensitive material. The unique surface microstructure, wrinkle microstructure, and internal hollow holes of the sensing layer contributed to its outstanding detection performance, including high sensitivity (335 kPa^–1^, 0.8–150 kPa), wide linear range (>500 kPa), low detection limit (0.8 kPa), and excellent long-lasting durability (>5000 cycles). Practical applications of the sensor were demonstrated, encompassing body joint motion recognition, real-time monitoring of subtle movement, and circuit switching control through voltage regulation to provide an intuitive means of monitoring pressure. This strategy provides a robust and efficient alternative to the development of new advanced materials and position the rGO-PDMS sponge-based pressure sensor a promising candidate for a wide range of technological applications. Research is still ongoing to introduce more responsive polymers to functionalize GO for the development of sensing materials for wearable devices.

## Supporting Information Available

Additional experimental details including sample synthesis, structure and characterizations, and sensor characterization methods (PDF)
