# Supplementary material for: Facile Graphene Oxide Modification Method via Hydroxyl-yne Click Reaction for Ultrasensitive and Ultrawide Monitoring Pressure Sensors
Source: ACS Appl Mater Interfaces. 2024 Jan 26;16(5):6198–207. doi: 10.1021/acsami.3c17172 (PMC10859893; doi:10.1021/acsami.3c17172)
Supplement: Supplementary file 1 — am3c17172_si_001.pdf [file am3c17172_si_001.pdf]

Supporting Information

A Facile Graphene Oxide Modification Method via Hydroxyl-yne Click Reaction for Ultra-sensitive and Ultra-wide Monitoring Pressure Sensors

Zhipeng Hu[1,2], Wanlong Lu[1], Youbin Zheng[2,3], Jiamei Liu[4], Hossam Haick[2], Laju Bu[1]**

[1]School of Chemistry, Xi'an Jiaotong University, Engineering Research Center of Energy Storage Materials and Devices, Ministry of Education, Xi'an Key Laboratory of Sustainable Energy Material Chemistry, Xi'an, Shaanxi 710049, P. R. China

[2]Department of Chemical Engineering and Russell Berrie Nanotechnology Institute, Technion-Israel Institute of Technology, Haifa 3200003, Israel

[3]Department of Electrical Engineering and Electronics, University of Liverpool, Liverpool L69 3GJ, United Kingdom

[4]Instrumental Analysis Center, Xi’an Jiaotong University, Xi'an, Shaanxi 710049, P. R. China

*Corresponding Author: laju2014@xjtu.edu.cn; hhossam@technion.ac.il

Materials: GO (Hangzhou Gaoxi Technology Co. Ltd., D50 < 10 μm), Propiolic acid (Alfa, 98%), Dodecyl alcohol (Sigma-Aldrich, 98%), Tetraethyleneglycol monomethyl ether (Sigma-Aldrich, 98%), Polyethylene glycol monomethylether (Alfa, M.W.5000), (3-Aminopropyl) triethoxysilane (Sigma-Aldrich, 98%), PDMS main agent and curing agent (Dow Corning Corporation, Sylgard 184). All chemicals were not further processed.

General procedure A (Preparation of propiolate esters) In a round-bottom flask, propargyl acid **1** (10 mmol), hydroxyl substrate **2** (11 mmol), solvent toluene (40 mL), and catalyst p-toluenesulfonic acid (1 mmol) were added sequentially. The reaction was then refluxed for 8 hours. After completion of the reaction, the target product propiolate esters **3** was purified by column chromatography or precipitation.

General procedure B (hydroxyl-yne click reaction) First, GO was dispersed in DMF (5 mg/mL) with the assistance of sonication. Then, the catalyst DABCO (20%) was added, followed by the dropwise addition of propiolate esters **3** in batches. The reaction was then stirred for 3 h at room temperature. The mixture was dialyzed by water or centrifuged to yield the modified GO-P.

Preparation of a PDMS Sponge: First, the PDMS main agent and curing agent were mixed at a mass ratio of 10:1, then the template cube sugar was immersed, vacuum pumped for 1 hour to remove air bubbles, and then the samples were cured at 80 °C for 3 h. The cube sugar was subsequently removed using hot water at 100 °C, and the resulting material was dried in a vacuum oven at 60 °C for 3 h to obtain PDMS sponge.

Preparation of a rGO-PDMS Sponge: The prepared PDMS sponge was treated with plasma (oxygen, 10 SCCM, 1 min). Then immersed into an ethanol solution of APTES (5 mmol/mL) for 2 h at room temperature. The modified sponge was washed with DDW and dried in a vacuum oven at 60 °C for 3 h. The modified PDMS sponge was immersed in GO-P2/DDW dispersion (2 mg/mL)

for 2 h. After the dip-coating process, the sponge was dried at 60 °C for 5 h to produce the GO-PDMS sponge. Subsequently, it underwent reduction using hydrazine hydrate vapor at 70 °C for 2 h, followed by washing with DDW and drying to yield the rGO-PDMS sponge.

Preparation of a rGO-PDMS Sponge based piezoresistive sensor: Gold interdigital electrodes were prepared on polyimide films by shadow-mask vaporization. The rGO-PDMS sponge sensing layer was placed on the electrode and two copper wires were connected by conductive silver paste. Finally, after packaging with PE film, rGO-PDMS sponge based piezoresistive sensor was successfully prepared. The dimensions of the rGO-PDMS sponge are 10mm * 10mm * 2.5mm (length * width * thickness). The specific parameters of the interdigital electrode are illustrated in Figure S6.

Characterizations and Measurements: The morphology and composition of the samples were characterized using scanning electron microscopy (SEM, Gemini SEM 500), X-ray diffraction spectroscopy (XRD, Bruker D8 ADVANCE), X-ray photoelectron spectroscopy (XPS, Thermo Fisher ESCALAB Xi+), Fourier-transform infrared spectrometer (FTIR, Nicolet iS10), optical contact angle measurement instrument (KRUSS DSA100S), and simultaneous thermal analyzer (TGA, METTLER TOLEDO TGA/DSC3+). Pressure and bending were loaded using a homemade automation module, and the current or voltage signals from the sensors were recorded by a digital electrometer (Agilent B2900A).

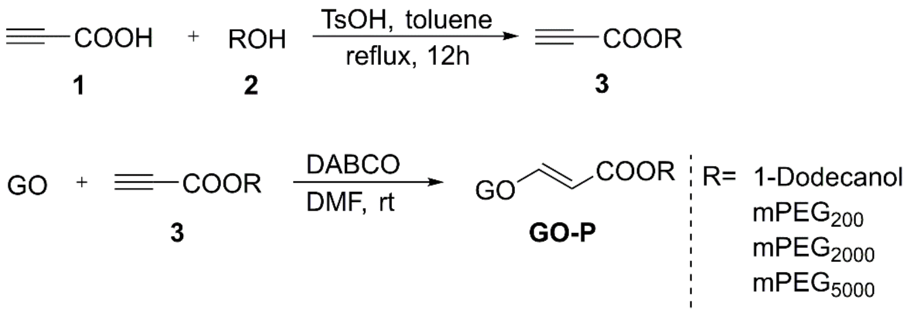

Scheme S1. Synthetic route for the functionalization of **GO** with a series of propiolates through a hydroxyl-yne click reaction.

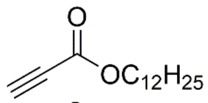

3a

3a was synthesized according to **General procedure A** using dodecanol. The residue was purified by column chromatography (silica gel, EtOAc/Petroleum ether) to afford the title compound (99% yield) as a colorless liquid. ^{1}H NMR (400 MHz, $CDCl_3$) δ 4.19 (t, *J* = 6.7 Hz, 2H), 2.87 (s, 1H), 1.67 (m, 2H), 1.47 – 1.14 (m, 18H), 0.88 (m, 3H). ^{13}C NMR (101 MHz, $CDCl_3$) δ 152.83, 74.80, 74.39, 66.49, 31.92, 29.63, 29.55, 29.47, 29.35, 29.17, 28.31, 25.75, 22.69, 14.11.

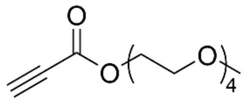

3b

3b was synthesized according to **General procedure A** using Tetraethyleneglycol monomethyl ether. The residue was purified by column chromatography (silica gel, EtOAc/Petroleum ether) to afford the title compound (99% yield) as a colorless liquid. ^{1}H NMR (400 MHz, $CDCl_3$) δ 4.42 – 4.30 (m, 2H), 3.81 – 3.70 (m, 2H), 3.71 – 3.60 (m, 10H), 3.59 – 3.51 (m, 2H), 3.38 (s, 3H), 2.92 (s, 1H). ^{13}C NMR (101 MHz, $CDCl_3$) δ 152.60, 75.47, 74.48, 71.85, 70.59, 70.55, 70.52, 70.50, 70.42, 68.48, 65.17, 58.93.

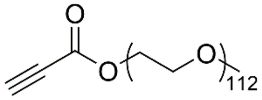

3c

3c was synthesized according to **General procedure A** using Polyethylene glycol monomethylether 5000. The polymer was precipitated into cold diethyl ether, and vacuum dried, yielding target product as a white solid. ^{1}H NMR (400 MHz, Chloroform-*d*) δ 4.59 – 4.23 (m, 2H), 3.65 (s, 446H), 3.38 (s, 3H), 2.98 (s, 1H). ^{13}C NMR (101 MHz, $CDCl_3$) δ 152.61, 75.75, 74.47, 72.51, 71.87, 70.63, 70.59, 70.51, 70.45, 70.26, 68.49, 65.15, 61.59, 58.98.

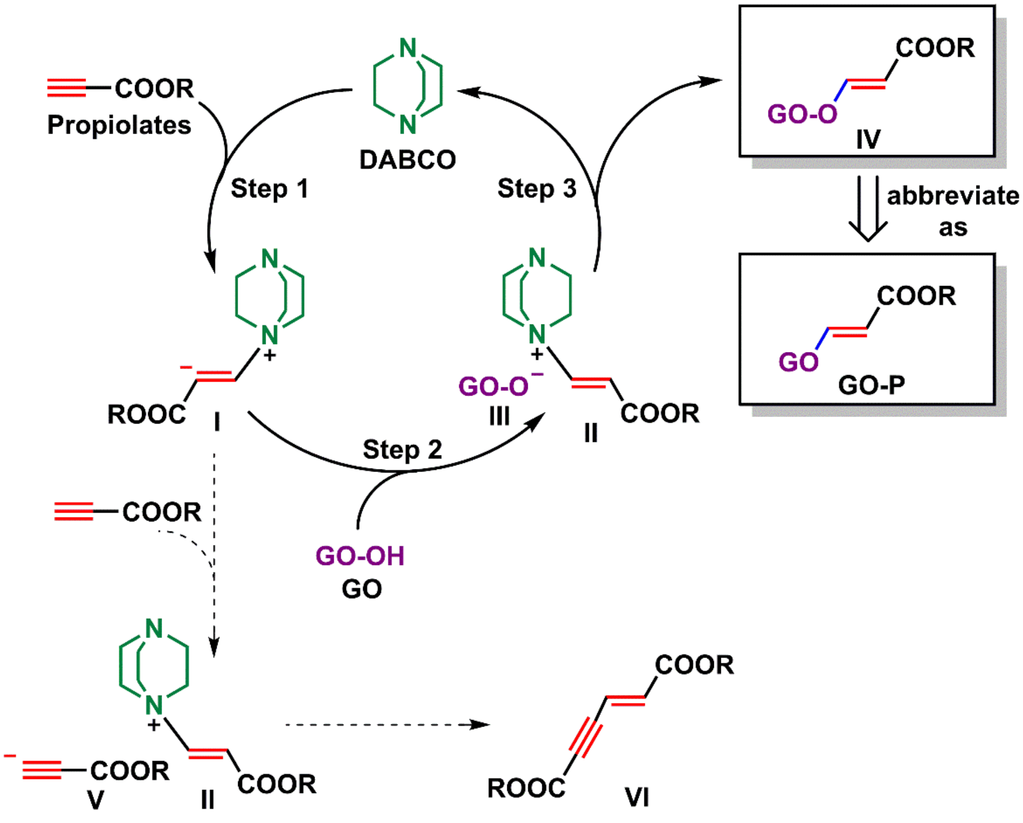

Scheme S2. Accepted mechanistic proposal for the Lewis-base (DABCO) -catalysed hydroxyl-yne click reaction.

Over the years, efforts have contributed to providing the most accepted mechanistic proposal for the Lewis-base (DABCO) -catalysed hydroxyl-yne click reaction.[1,2] In this mechanism, the reaction is divided into three steps as shown in **Scheme S2**. In step 1, the catalyst (DABCO) nucleophilically attacks the triple bond to form a more basic zwitterion **I**. In step 2, zwitterion **I** reacts with GO to generate intermediates **II** and **III** (deprotonation of hydroxyl groups on GO). In step 3, **III** nucleophilically attacks intermediate **II**, undergoing nucleophilic substitution to yield the final product **GO-P** and the catalyst DABCO. The DABCO continues to participate in the catalytic cycle. It should be noted that step 1 is the fast step of the reaction. There is a competing reaction in step 2, as indicated by the dashed arrow, where zwitterion I might undergo nucleophilic substitution with the propiolates to generate by-product **VI**. This process can be effectively suppressed by reducing the concentration of the propiolates in the reaction system, which can be achieved by gradually adding them in batches, as outlined in General procedure B. Additionally, it should be noted that the by-product can be easily removed by centrifugation.

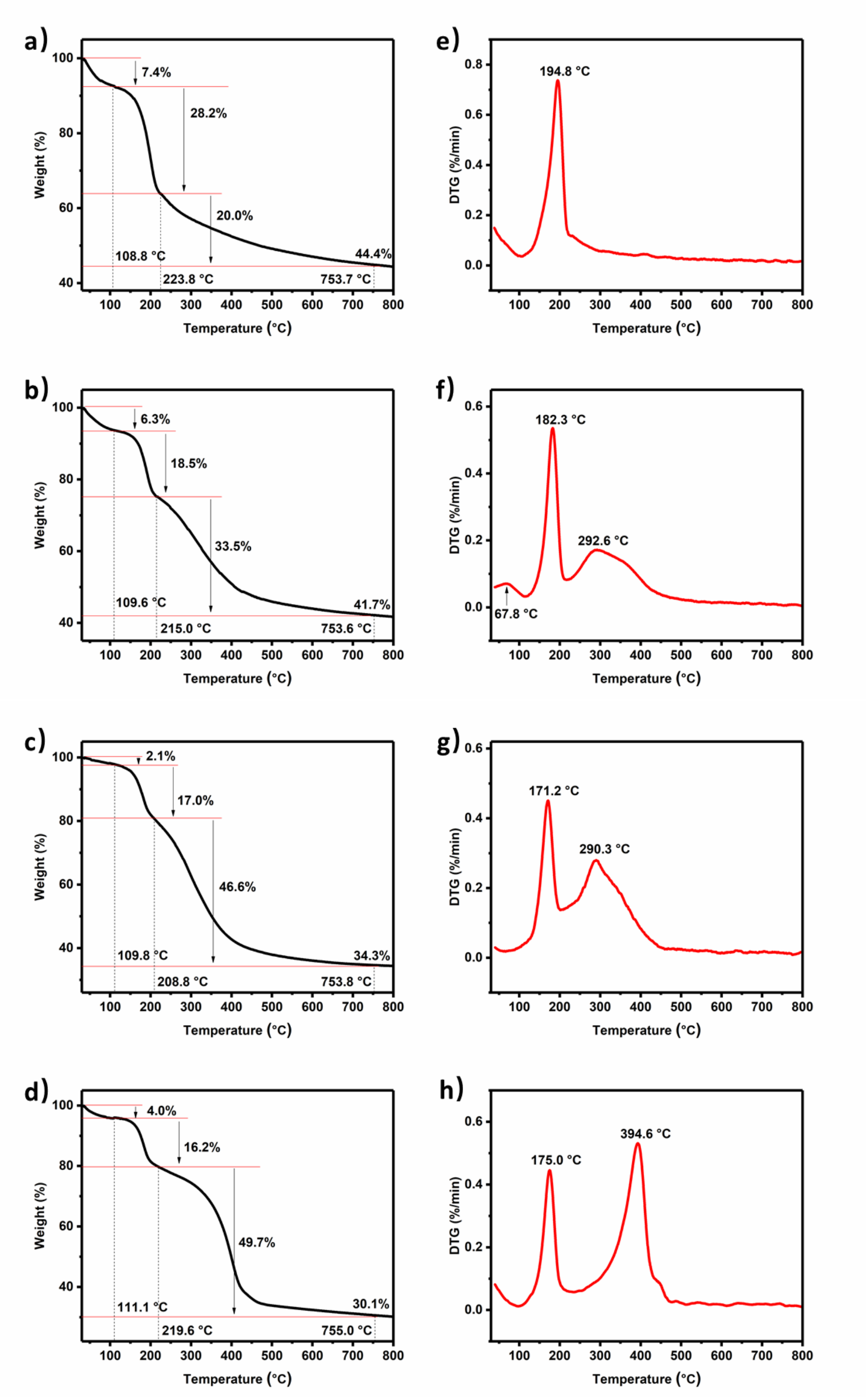

Figure S1. (a-d) TGA curves and (e-h) DTG curves of GO, GO-P1, GO-P2 and GO-P3, respectively.

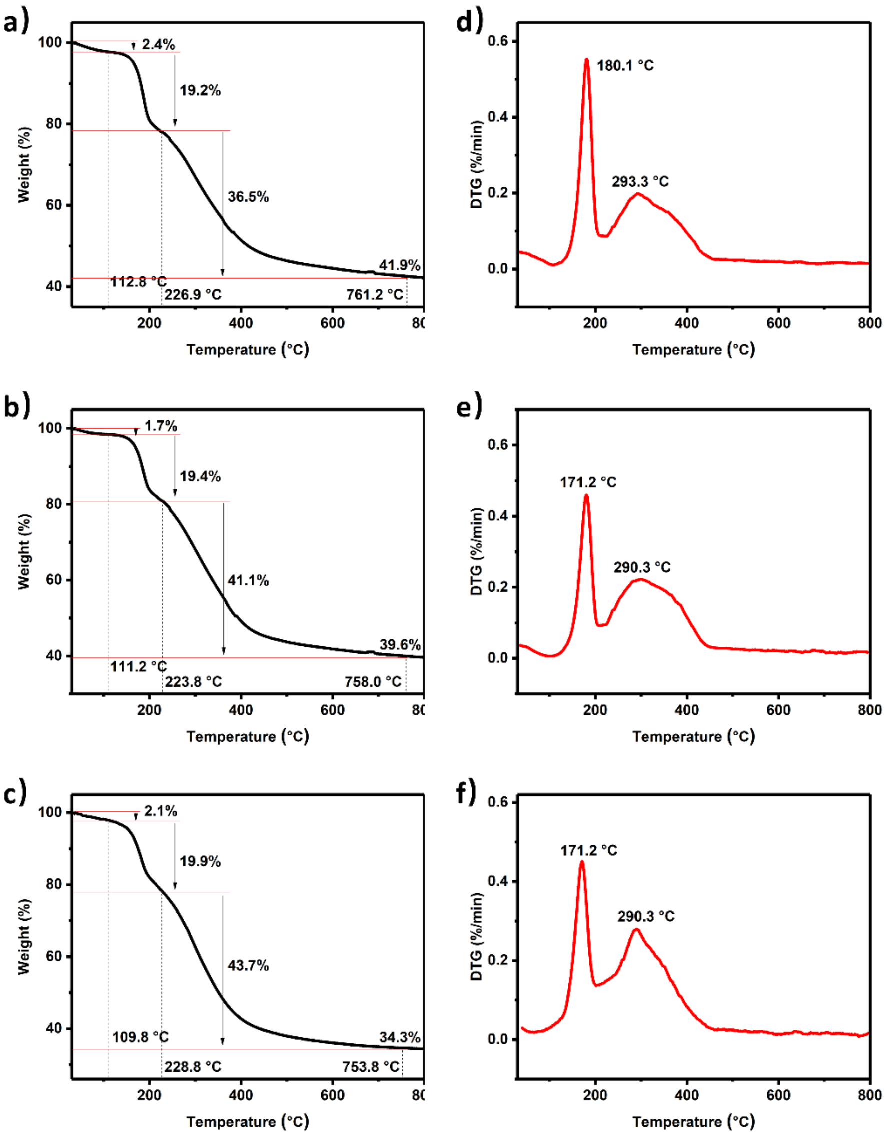

Figure S2. (a-c) TGA curves and (d-f) DTG curves for GO-P2 with grafting ratios of 2.5%, 4.8%, and 10.1%, respectively.

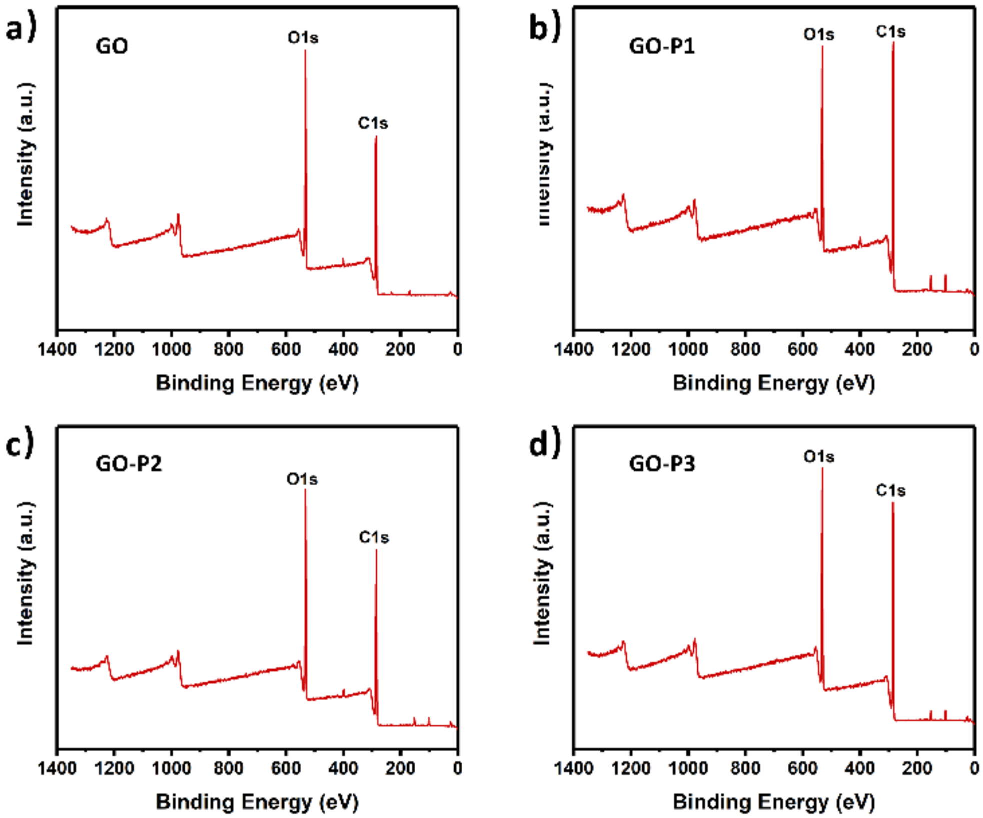

e)

	GO	GO-P1	GO-P2	GO-P3
C (Atomic %)	70.52	76.70	71.35	72.78
O (Atomic %)	29.48	23.30	28.65	27.22

Figure S3. Survey-mode XPS spectra of (a) GO, (b) GO-P1, (c) GO-P2, (d) GO-P3, and (e) the corresponding component analysis.

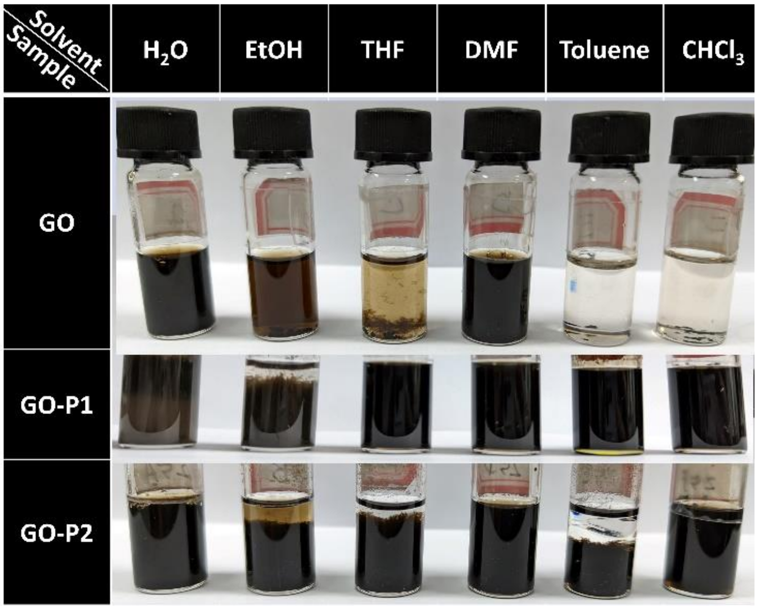

Figure S4. Dispersion of GO, hydrophobic modified GO-P1 and hydrophilic modified GO-P2 in various solvents (concentration of 1 mg/mL, images were taken immediately after preparation).

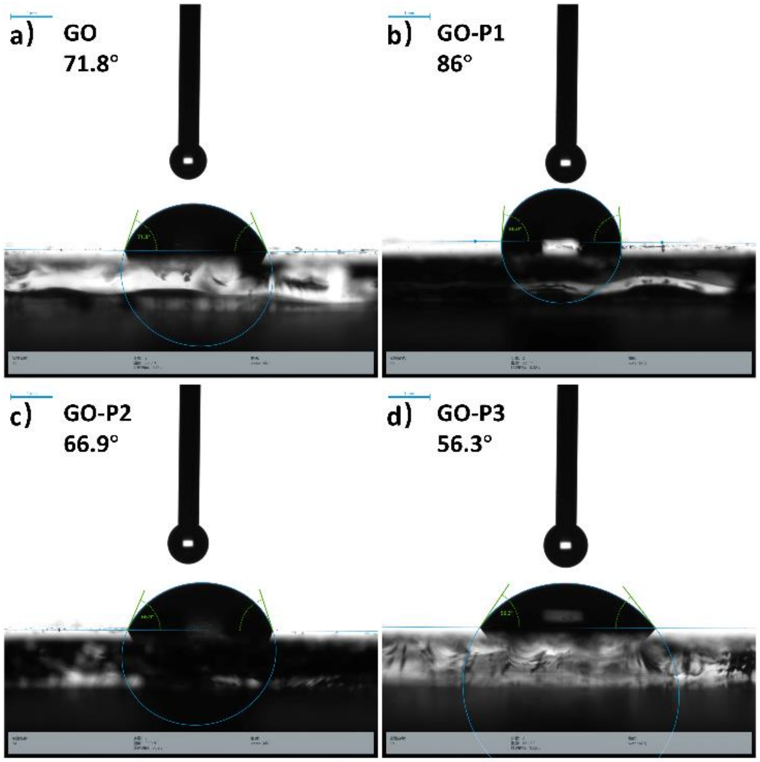

Figure S5. Optical images of the contact angles of GO, GO-P1, GO-P2 and GO-P3.

Water droplet images and water contact angle measurements were presented. The contact angle of GO was measured at 71.8°. After modification with the hydrophobic dodecyl propiolate, the contact angle of GO-P1 increased to 86°. On the other hand, after the modification with the hydrophilic mPEG ester, the contact angles of GO-P2 and GO-P3 decreased to 66.9° and 56.3° respectively. Notably, the contact angle difference between GO-P1 and GO-P3 in comparison to GO exceeded 10°, indicating a substantial alteration in the hydrophilic/hydrophobic nature of GO by graft modification. These results were consistent with the TGA and FTIR data, providing further confirmation of the successful hydroxyl-yne click modification.

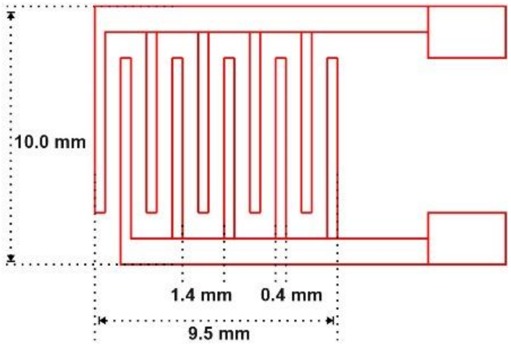

Figure S6. Parameter schematic of the interdigital electrode.

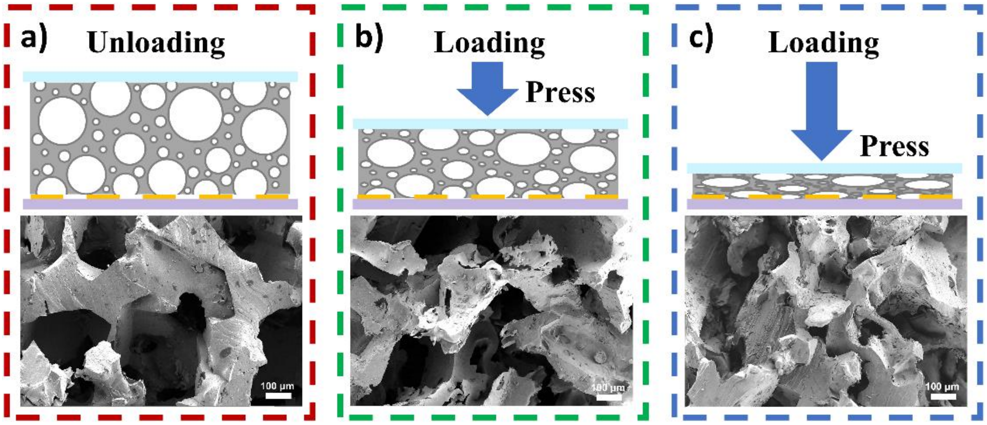

Figure S7. Schematic illustration of the sensing mechanism of the rGO-PDMS sponge based piezoresistive sensor and SEM images of the sensing layer (a) in the initial state, (b) under slight pressure, and (c) under high pressure, respectively.

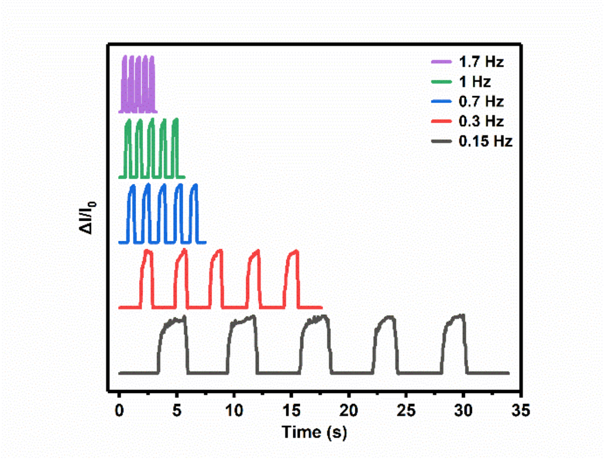

Figure S8. The I–T curves of the sensor under 8 kPa pressure with different driving frequencies.

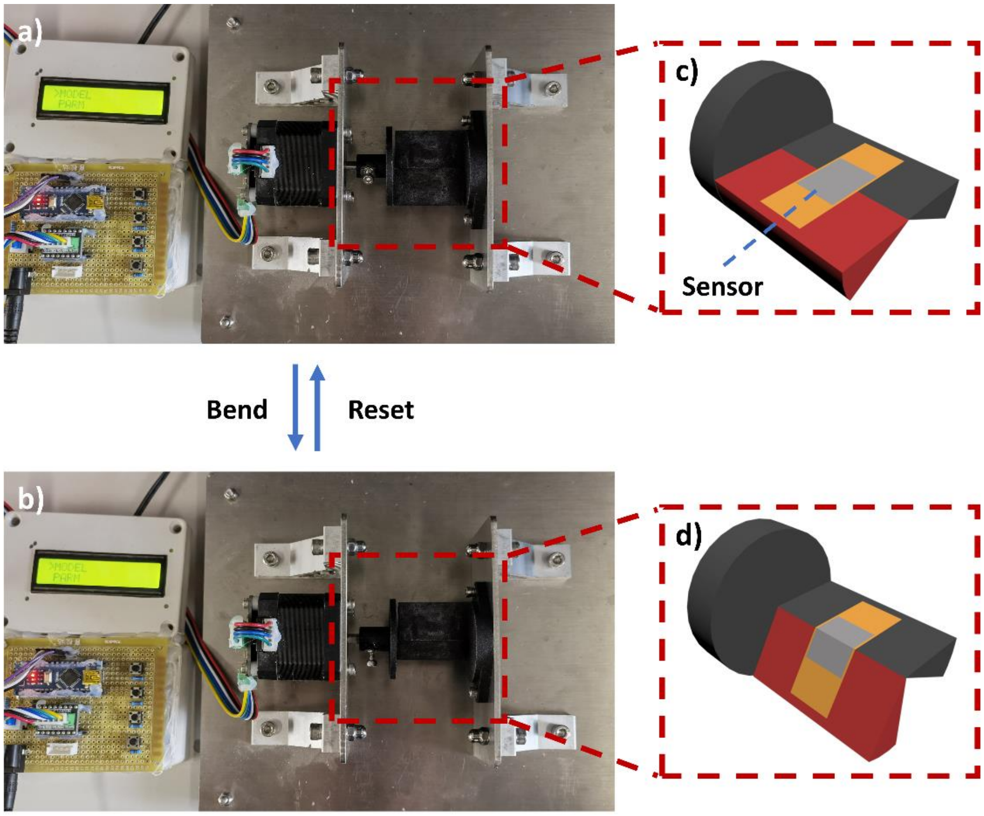

Figure S9. Photographs and schematics of a homemade bending test device: (a) and (c) bending, (b) and (d) resetting states.

In this study, the bending tests were conducted using a homemade device. As illustrated in Figure S9, bending and resetting are achieved through the relative motion of two designed

triangular prisms. Specifically, the sensor is fixed on a platform formed by two closely positioned prisms. The black prism is affixed to the substrate, while the red prism, driven by a motor, reciprocally rotates around the central axis, thereby achieving the bending and resetting of the device. Ideally, the rotation center here is a corner, resulting in a radius of curvature that approaches zero.

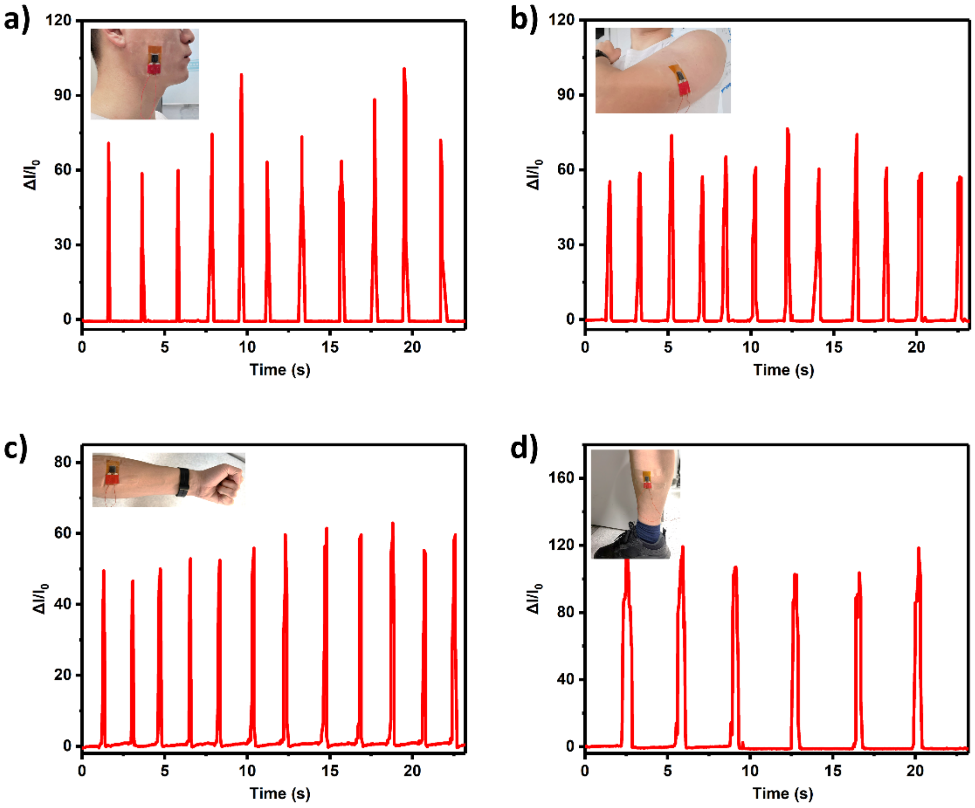

Figure S10. Practical applications of the rGO-PDMS sponge based piezoresistive sensor for monitoring human (a) masticatory muscle, (b) triceps brachii muscle, (c) forearm extensor, and (d) tibialis anterior muscle movement.

Table S1. Comparison of this work with other reported flexible pressure sensors

Active materials	Sensitivity (kPa^{-1})	Measurement range (kPa)	Durability	Ref.
rGO-PDMS sponge	335<br>41	0.8-150<br>150-500	5000	This work
Graphene-PDMS sponge	17.9	0-30	10000	3
Graphene-[a)]PU sponge	7.62	0-50	1000	4
[b)]PPy/rGO-PU sponge	0.635	0-30	3000	5
[c)]N-GNS/ [d)]LM/PDMS sponge	476	0-3.4	10000	6
Sparkling graphene block ([e)]TWEEN 80)	229.8<br>26.86	0-0.1<br>0.4-1	1000	7
Graphene-PDMS micropillar	1.2	0-25	1000	8
Graphene-PDMS micropillar	10.41	0-2.5	10000	9
MXene sponge	147<br>442	0-5.37<br>5.37-18.56	10000	10
MXene-P(VDF-TrFE) film	817.4<br>2213.68	0.072-0.74<br>0.74-3.083	-	11
Dual-microstructured MXene film	401.01<br>16.026	0.002-12<br>12-100	6000	12
AgNW micropillar	128.29<br>1.28	0-0.2<br>0.2-10	10000	13
[f)]CPDMS/ [g)]AgNW [h)]DCL	3788.29	0-6	22000	14
[i)]CNT bundles	141.72<br>18.76	0-40<br>40-100	1000	15
CNT-PDMS sponge	0.3<br>0.9	0-1<br>15-50	-	16
Graphene-PDMS sponge	0.12<br>0.042<br>0.004	0-10<br>10-100<br>100-500	5000	17
[j)]GNPs/[k)]MWCNT-PU sponge	0.062	0-4.30	2000	18
rGO-melamine foam	0.108	0.013-15	2000	19
[l)]GPN-PDMS foam	0.09<br>0.0067	0.09-1000<br>1200-1800	-	20
MWCNT-rGO-PU foam	0.022<br>0.088<br>0.034	0-2.7<br>2.7-10.8<br>10.8-48.8	5000	21
MWCNT-PDMS foam	2.155<br>0.063<br>0.022	0.05-2<br>2-200<br>200-500	2500	22

Notes: a) PU: Polyurethane; b) PPy: Polypyrrole; c) N-GNS: Nitrogen-doped graphene nanosheets; d) LM: Liquid metal; e) TWEEN 80: Polyethylene glycol sorbitan monooleate; f) CPDMS: Carbon black/polydimethylsiloxane; g) AgNW: Silver Nanowire; h) DCL: Double

conductive layer; i) CNT: Carbon nanotube; j) GNPs: Graphene nanoplatelets; k) MWCNT: multiwalled carbon nanotubes; l) GPN: Graphene porous network.

Table S2. Unloading current of the sensors for different test scenarios.

Current (A) / Time (s)	Pressure unloading-loading test (4 kPa)	Pressure unloading-loading test (24 kPa)	Pressure unloading-loading test (380 kPa)	Bending test (45°)	Finger test
9.34	1.81E-11	4.01E-11	2.35E-10	1.88E-06	2.21E-07
9.37	1.49E-11	5.07E-11	3.02E-10	6.39E-07	1.66E-07
9.41	2.98E-11	2.96E-11	2.14E-10	5.27E-07	1.69E-07
9.44	2.86E-11	4.34E-11	4.41E-10	4.90E-07	1.66E-07
9.47	1.09E-11	6.64E-11	1.42E-10	4.38E-07	1.49E-07
9.51	1.31E-11	8.94E-11	4.21E-11	3.13E-07	1.30E-07
9.55	1.80E-11	1.16E-10	7.18E-11	1.58E-07	5.83E-08
9.58	1.14E-11	1.47E-10	2.06E-11	5.06E-08	1.78E-08
9.63	2.64E-11	1.96E-10	1.06E-11	3.12E-08	1.36E-08
9.66	3.36E-11	2.87E-10	8.00E-11	2.94E-08	8.81E-09
9.69	3.20E-11	1.89E-10	4.52E-11	1.58E-08	7.13E-09
9.72	1.71E-11	2.08E-10	-1.20E-11	6.69E-08	1.06E-07
9.76	3.85E-10	2.31E-10	1.56E-11	1.81E-07	2.84E-07
9.81	1.51E-10	5.75E-11	1.76E-11	4.31E-07	3.37E-07
9.85	4.89E-11	5.23E-11	9.04E-11	4.59E-07	5.91E-07
9.88	1.01E-10	2.41E-11	9.89E-11	5.09E-07	7.28E-07
9.91	1.03E-10	2.14E-10	5.36E-11	7.75E-07	1.27E-06
9.94	9.71E-11	1.03E-10	1.88E-11	1.24E-06	2.06E-06

Notes: Unloading current: marked red.

The initial current (unloading current) is a crucial parameter that is not only associated with the sensor itself but also closely linked to the testing scenarios. For the pressure unloading-loading test, which approximates an ideal testing scenario primarily related to the intrinsic performance of the sensor, the typical actual unloading current is around 10^{-10}. When calculating sensitivity, we use a value of $1*10^{-9}$ as the unloading current to eliminate the influence of noise and ensure more

reliable results. For bending and wearable applications tests, to eliminate noise interference (especially off-design motions of the sensor), it is common practice to fix the sensor onto a substrate. However, this fixation may subject the sensor to a certain level of initial pressure, resulting in an increase in the initial current.

References

(1) Tejedor, D.; Álvarez-Méndez, S. J.; López-Soria, J. M.; Martín, V. S.; García-Tellado, F. A Robust and General Protocol for the Lewis-Base-Catalysed Reaction of Alcohols and Alkyl Propiolates. *Eur. J. Org. Chem.* **2014**, *2014*, 198.

(2) Li, B.; Xu, C.; Yu, J.; Liu, L.; Zhang, X.; Fan, Y. One-Pot Cellulose Etherification and Self-Crosslinking Via a Mild Hydroxyl–Yne Click Reaction in a Homogeneous System. *Green Chem.* **2023**, *25*, 2608.

(3) Kaiqiang, W.; Xingyang, L. Wearable Pressure Sensor for Athletes' Full-Range Motion Signal Monitoring. *Materials Research Express* **2020**, *7*, 105003.

(4) Feng, C.; Yi, Z.; Jin, X.; Seraji, S. M.; Dong, Y.; Kong, L.; Salim, N. Solvent Crystallization-Induced Porous Polyurethane/Graphene Composite Foams for Pressure Sensing. *Composites Part B: Engineering* **2020**, *194*, 108065.

(5) Wang, R.; Tan, Z.; Zhong, W.; Liu, K.; Li, M.; Chen, Y.; Wang, W.; Wang, D. Polypyrrole (Ppy) Attached on Porous Conductive Sponge Derived from Carbonized Graphene Oxide Coated Polyurethane (Pu) and Its Application in Pressure Sensor. *Compos. Commun.* **2020**, *22*, 100426.

(6) Li, Y.; Cui, Y.; Zhang, M.; Li, X.; Li, R.; Si, W.; Sun, Q.; Yu, L.; Huang, C. Ultrasensitive Pressure Sensor Sponge Using Liquid Metal Modulated Nitrogen-Doped Graphene Nanosheets.

Nano Letters **2022**, *22*, 2817.

(7) Lv, L.; Zhang, P.; Xu, T.; Qu, L. Ultrasensitive Pressure Sensor Based on an Ultralight Sparkling Graphene Block. *ACS Appl. Mater. Interfaces* **2017**, *9*, 22885.

(8) Shi, J.; Wang, L.; Dai, Z.; Zhao, L.; Du, M.; Li, H.; Fang, Y. Multiscale Hierarchical Design of a Flexible Piezoresistive Pressure Sensor with High Sensitivity and Wide Linearity Range. *Small* **2018**, *14*, 1800819.

(9) Cheng, L.; Qian, W.; Wei, L.; Zhang, H.; Zhao, T.; Li, M.; Liu, A.; Wu, H. A Highly Sensitive Piezoresistive Sensor with Interlocked Graphene Microarrays for Meticulous Monitoring of Human Motions. *J. Mater. Chem.* **2020**, *8*, 11525.

(10) Yue, Y.; Liu, N.; Liu, W.; Li, M.; Ma, Y.; Luo, C.; Wang, S.; Rao, J.; Hu, X.; Su, J.; Zhang, Z.; Huang, Q.; Gao, Y. 3d Hybrid Porous Mxene-Sponge Network and Its Application in Piezoresistive Sensor. *Nano Energy* **2018**, *50*, 79.

(11) Li, L.; Fu, X.; Chen, S.; Uzun, S.; Levitt, A. S.; Shuck, C. E.; Han, W.; Gogotsi, Y. Hydrophobic and Stable Mxene–Polymer Pressure Sensors for Wearable Electronics. *ACS Appl. Mater. Interfaces* **2020**, *12*, 15362.

(12) Guo, L.; Li, Z.; Hu, W.; Liu, T.; Zheng, Y.; Yuan, M.; Dai, Y.; Ning, R.; Zhu, Y.; Tao, K.; Zhang, M.; Du, T.; Zhang, L.; Su, C.; Haick, H.; Wu, W. A Flexible Dual-Structured Mxene for Ultra-Sensitive and Ultra-Wide Monitoring of Anatomical and Physiological Movements. *J. Mater. Chem. A* **2021**, *9*, 26867.

(13) Ji, B.; Mao, Y.; Zhou, Q.; Zhou, J.; Chen, G.; Gao, Y.; Tian, Y.; Wen, W.; Zhou, B. Facile Preparation of Hybrid Structure Based on Mesodome and Micropillar Arrays as Flexible Electronic Skin with Tunable Sensitivity and Detection Range. *ACS Appl. Mater. Interfaces* **2019**, *11*, 28060.

(14) Ji, B.; Zhou, Q.; Wu, J.; Gao, Y.; Wen, W.; Zhou, B. Synergistic Optimization toward the

Sensitivity and Linearity of Flexible Pressure Sensor Via Double Conductive Layer and Porous Microdome Array. *ACS Appl. Mater. Interfaces* **2020**, *12*, 31021.

(15) Sim, S.; Jo, E.; Kang, Y.; Chung, E.; Kim, J. Highly Sensitive Flexible Tactile Sensors in Wide Sensing Range Enabled by Hierarchical Topography of Biaxially Strained and Capillary-Densified Carbon Nanotube Bundles. *Small* **2021**, *17*, 2105334.

(16) Iglio, R.; Mariani, S.; Robbiano, V.; Strambini, L.; Barillaro, G. Flexible Polydimethylsiloxane Foams Decorated with Multiwalled Carbon Nanotubes Enable Unprecedented Detection of Ultralow Strain and Pressure Coupled with a Large Working Range. *ACS Appl. Mater. Interfaces* **2018**, *10*, 13877.

(17) Kou, H.; Zhang, L.; Tan, Q.; Liu, G.; Dong, H.; Zhang, W.; Xiong, J. Wireless Wide-Range Pressure Sensor Based on Graphene/Pdms Sponge for Tactile Monitoring. *Scientific Reports* **2019**, *9*, 3916.

(18) Qiu, J.; Guo, X.; Chu, R.; Wang, S.; Zeng, W.; Qu, L.; Zhao, Y.; Yan, F.; Xing, G. Rapid-Response, Low Detection Limit, and High-Sensitivity Capacitive Flexible Tactile Sensor Based on Three-Dimensional Porous Dielectric Layer for Wearable Electronic Skin. *ACS Appl. Mater. Interfaces* **2019**, *11*, 40716.

(19) Yu, T.; Tao, Y.; Wu, Y.; Zhang, D.; Yang, J.; Liu, X. Self-Encapsulated Breathable Reduced Oxide Graphene-Coated Melamine Foam for Stress Sensing. *ACS Appl. Electron. Mater.* **2023**, *5*, 4624.

(20) Pang, Y.; Tian, H.; Tao, L.; Li, Y.; Wang, X.; Deng, N.; Yang, Y.; Ren, T. L. Flexible, Highly Sensitive, and Wearable Pressure and Strain Sensors with Graphene Porous Network Structure. *ACS Appl. Mater. Interfaces* **2016**, *8*, 26458.

(21) Tewari, A.; Gandla, S.; Bohm, S.; McNeill, C. R.; Gupta, D. Highly Exfoliated Mwnt–Rgo

Ink-Wrapped Polyurethane Foam for Piezoresistive Pressure Sensor Applications. *ACS Appl. Mater. Interfaces* **2018**, *10*, 5185.

(22) Feng, Z.; He, Q.; Wang, X.; Lin, Y.; Qiu, J.; Wu, Y.; Yang, J. Capacitive Sensors with Hybrid Dielectric Structures and High Sensitivity over a Wide Pressure Range for Monitoring Biosignals. *ACS Appl. Mater. Interfaces* **2023**, *15*, 6217.
